# Electrochemical Detection of Neurotransmitters

**DOI:** 10.3390/bios10080101

**Published:** 2020-08-18

**Authors:** Saikat Banerjee, Stephanie McCracken, Md Faruk Hossain, Gymama Slaughter

**Affiliations:** Frank Reidy Research Center for Bioelectrics, Department of Electrical and Computer Engineering, Old Dominion University, Norfolk, VA 23508, USA; sbane001@odu.edu (S.B.); smccr007@odu.edu (S.M.); fhossain@odu.edu (M.F.H.)

**Keywords:** neurotransmitters, electrochemical, biosensors, fast scan cyclic voltammetry, differential pulse voltammetry, cyclic voltammetry

## Abstract

Neurotransmitters are important chemical messengers in the nervous system that play a crucial role in physiological and physical health. Abnormal levels of neurotransmitters have been correlated with physical, psychotic, and neurodegenerative diseases such as Alzheimer’s, Parkinson’s, dementia, addiction, depression, and schizophrenia. Although multiple neurotechnological approaches have been reported in the literature, the detection and monitoring of neurotransmitters in the brain remains a challenge and continues to garner significant attention. Neurotechnology that provides high-throughput, as well as fast and specific quantification of target analytes in the brain, without negatively impacting the implanted region is highly desired for the monitoring of the complex intercommunication of neurotransmitters. Therefore, it is crucial to develop clinical assessment techniques that are sensitive and reliable to monitor and modulate these chemical messengers and screen diseases. This review focuses on summarizing the current electrochemical measurement techniques that are capable of sensing neurotransmitters with high temporal resolution in real time. Advanced neurotransmitter sensing platforms that integrate nanomaterials and biorecognition elements are explored.

## 1. Introduction

A neurotransmitter (NT) is a chemical messenger that influences a wide variety of both psychological and physiological functions. Their functions are directly linked to the central nervous system that controls the human body and directly supervise neurophysiological processes such as sleep, emotions, memory, and other cognitive functions [1,2]. Abnormalities in the concentration and dysfunction of NTs in the central nervous system have been linked to a multitude of diseases such as psychotic (schizophrenia, depression, dementia, etc.), neurodegenerative diseases (Alzheimer’s, Parkinson’s, Huntington’s disease, autism, epilepsy, attention deficit hyperactivity disorder, etc.), and other illnesses (glaucoma, arrhythmias, thyroid hormone shortage, congestive heart damage, sudden infant death syndrome, dejection and anguish, etc.) [3,4]. German pharmacologist Otto Loewi was the first to discover the most widely detected neurotransmitter, acetylcholine in 1921. The exact number of unique NTs in humans is unknown, but more than 200 have been identified since 1921 [2].

NTs are classified according to their functions (excitatory and inhibitory), molecular structures (amino acid group, biogenic amines, and soluble gases) and action (fast acting and slow acting). Most NTs are difficult to classify according to the above categories. For example, dopamine (DA) can be classified as excitatory and inhibitory, and acetylcholine and choline can be classified by other molecular groups [5], hence NTs are generally classified according to their molecular structure, such as amino acid based NTs (e.g., glutamic acid, tryptophan, Gamma-Aminobutyric Acid (GABA), L- aspartate, D-serine, glycine, histidine, and tyrosine), biogenic amines (e.g., acetylcholine, serotonin, histamine, epinephrine, norepinephrine, and dopamine), and soluble gases (e.g., nitric oxide and hydrogen sulfide) [2,6]. Glutamate, histamine, and acetylcholine are examples of NTs that bear non-electroactive properties and are difficult to measure without the presence of a biorecognition element such as an enzyme. The enzyme is selective towards its target NT, wherein it converts the biorecognition event into an analytical signal. The limitations of enzyme-based biosensors are their complex immobilization process and instability due to the degradation of enzymatic activities overtime. However, electroactive NTs can be easily detected using nonenzymatic sensors. Although these NTs biosensors are inexpensive, biocompatible, and stable for most applications, their specificity or selectivity remain questionable. Many biochemical species in the brain have similar oxidation–reduction potentials and the presence of many interfering species hinders the deployment of nonenzymatic NTs biosensors as these types of biosensors face difficulties in detecting specific target analytes in the brain.

Neurotransmitter analyses under in vitro and *in vivo* conditions are necessary for understanding the functioning of the brain and its disorders because of their importance in clinical assessments [7,8,9]. However, NT concentrations in biological samples are relatively low (nM) and therefore require highly sensitive, selective, and reliable biosensors to detect them. Several efforts have been employed to develop transduction mechanisms for the detection of NTs, such as nuclear medicine tomographic imaging (i.e., positron emission tomography and single-photon emission computed tomography), optical sensing (i.e., surface-enhanced Raman spectroscopy, fluorescence, chemiluminescence, optical fiber based biosensors, and colorimetry), analytical chemistry techniques (i.e., high performance liquid chromatography), and microdialysis [10]. These reported techniques require highly trained personnel and are time-consuming, thereby not suitable for point of care testing.

A rapid and accurate detection of NTs are key factors in point of care diagnostics for clinical and medical applications. Electrochemical transduction methods have received significant attention and are an ideal analytical approach for the characterization of biomolecules due to their low cost, ease of use, small-size, and fast response time, as well as the possibility to detect two or more analytes simultaneously [11,12]. In this review, we focus on electrochemical characterization techniques, such as cyclic voltammetry (CV) including fast scan cyclic voltammetry (FSCV) and differential pulse voltammetry (DPV) for the electrochemical sensing of glutamate (Glu), acetylcholine (AChE), dopamine (DA), and serotonin (5-hydroxytryptamine (5-HT)). Electrochemical techniques have significant advantages over conventional analytical techniques. They enable device miniaturization, fast response time, high sensitivity, and good selectivity while being particularly suited for the detection of neurotransmitters in the presence of mixtures of other biomolecules with high temporal resolution. The various advancements made in the development of electrochemical transducers for single and multiple analytes in vitro and *in vivo* are highlighted.

## 2. Biosensing of Neurotransmitters

Glutamate (L-glutamate acid) is a prominent amino acid neurotransmitter in the central nervous system [2] in that almost 90% of all neurons use this amino acid as a primary messenger molecule. The imbalance of glutamate concentrations through the neural pathways leads to neurological and psychiatric disorders like Alzheimer’s diseases, Parkinson’s disease, autism, depression, stroke, schizophrenia, and epilepsy. Therefore, being able to monitor changes in glutamate is valuable in studying these disorders. Acetylcholine (AChE) is one of the most studied neurotransmitters and it plays an important role in brain and muscle function. Imbalances in acetylcholine lead to neurological conditions, such as regulating physiological levels of different neurotransmitters, opening of ligand- gated ion channels, and the bursting mode of neuronal firing. Patients with Alzheimer’s disease, Parkinson’s disease, and myasthenia gravis exhibit low levels of acetylcholine and the concentrations of AChE vary in behavioral, learning, and sleep disorders [13].

Monamine neurotransmitters, dopamine and 5-hydroxytryptamine (5-HT) also know as serotonin are of great clinical significance for the role they play in motor functions, emotional responses, motivations, and behavioral functions. An imbalance of dopamine has been associated with physiological and psychological disorder. Increased dopaminergic activity may lead to errors in perception such as hallucinations or psychiatric diseases such as schizophrenia. Additionally, deficiency of dopaminergic neurons is often associated with neurological/motor diseases such as depression and Parkinson’s diseases. Serotonin is known to regulate mood, sleep patterns, appetite, body temperature, and hormonal activity. Some researchers hypothesize that low concentrations of serotonin contribute to disorders such as anxiety and depression.

**Figure 1:**
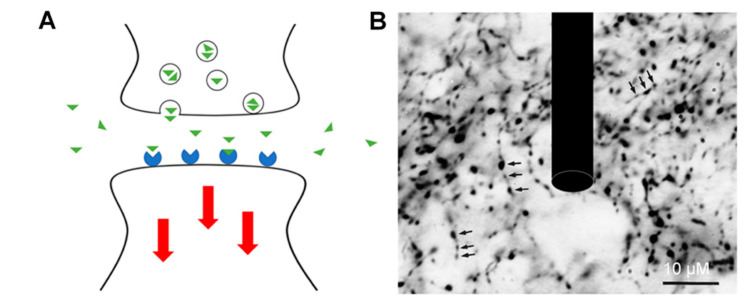
(**A**) Neurotransmitters (green) are exocytotically released into the synaptic cleft and diffuse into the extracellular matrix, bind to the receptors (blue), and trigger a series of downstream reactions (red) in the axon of other neurons. (**B**) A model electrode drawn to scale in a network of neurons. Adapted from [14].

Since NTs play critical roles in the central nervous system and impact the severity of neurodegenerative diseases, real-time detection of NTs is of great interest in neuroscience [14]. The human brain contains billions of neurons which communicate through trillions of synapses and electrical channels. A concentration gradient of NTs is formed between neurons when exocytotically released. The NTs act as ligands which reversibly bind to receptors on adjacent cells, causing a conformational change which triggers a series of biochemical responses within the cell. Due to the nanometer scale of the synapse and the micron scale of electrode probes, NT detection occurs in the extracellular space as shown in Figure 1. The quantification of NT continues to be a challenge for most conventional analytical methods due to the fast release and clearance of NT, low concentration levels, and the presence of interfering analytes in the extracellular space.

**Figure 2:**
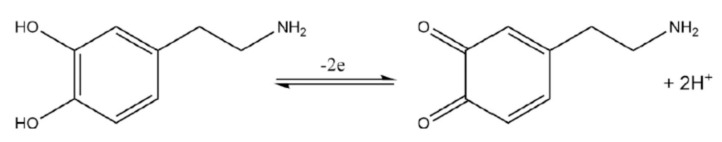
Electrochemical oxidation of dopamine to dopamine quinone in a 2-electron oxidation when a potential is applied to the transducer.

Electrochemical transducers are traditionally modified using different electrocatalytic materials and biorecognition elements (bioreceptors) to improve the sensitivity, selectivity, and reliability of the transducer. Conductive polymers (CPs) have been used to modify and improve the catalytic activity of the modified electrode surface via electrodeposition. The polymeric film modified transducer can detect NTs in the presence of interference species [15,16]. Although polymeric modified biosensors detect NTs, CPs are somewhat limited due to their low sensitivity and reliability because of the poor electron transfer between the target molecule and the current collector [16]. Carbon based materials, such as graphene, carbon nanotubes (CNTs), and carbon black activated carbon are widely used in the fabrication of electrochemical transducers due to their good physical and chemical properties, high conductivity, relative inertness, wide voltage range, and fast heterogeneous electron transfer [17,18]. For example, carbon microelectrodes have been used to directly detect and monitor the fast changes in monoamine neurotransmitters, such as dopamine. Figure 2 shows that the electrochemical detection of dopamine involves 2e^−^/2H^+^ oxidation–reduction (redox) reaction at an applied potential. The oxidation product is dopamine quinone and the current generated from the transfer of 2 electrons and the release of 2 protons is directly proportional to the concentration of dopamine. Thereby, enabling the electrochemical quantification of dopamine. The three primary types of electrochemical transduction methods (cyclic voltammetry, fast scan cyclic voltammetry, and differential pulsed voltametry) for NT detection are discussed in the subsequent sections.

### 2.1. Neurosensing via Cyclic Voltammetry

Cyclic voltammetry (CV) is an indispensable tool in electrochemistry and is used to study the electrocatalytic performance of electrodes, electrode sensing materials, and electron transfer involving NTs. Generally, a fixed voltage is applied to a three-electrode system consisting of a working, counter, and reference electrodes. Current flows between the working and counter electrode during a redox chemical reaction and the reference electrode maintains a constant potential between the electrodes. The positions, shapes of the redox peaks, and relative peak amplitudes generated in a cyclic voltammogram are determined by the electron transfer rate and chemical stability of the analyte(s). The oxidation peak generated is due to the oxidation of NT at the electrode surface and the amplitude of the oxidation peak increases corresponding to higher concentration of the NT while the concentration can be directly quantified using the peak current values. Considering these factors, CV has been explored as the method to detect electroactive neurotransmitters.

An electrochemical biosensor array in which a nanoporous pseudo carbon paste (nano-PPCPE) working electrode was developed for the simultaneous detection of glutamate and AChE [19]. The nano-PPCPE is comprised of a mixture of graphite powder, polystyrene microspheres, and polymerized pyrrole packed into glass tubes with a copper wire (Φ = 3 mm (inner)). The cyclic voltammogram generated at a scan rate of 0.05 V/s indicated two anodic peaks belonging to glutamate and AChE. Additionally, the sensor was capable of distinguishing between the two NTs, thereby implying a selective and simultaneous detection of glutamate and AChE is feasible for multimodal detection of NTs. A linear range of 0.5 μM—10 μM with the detection limit of 0.25 μM was demonstrated for glutamate, while a linear range of 5 μM—200 μM with the detection limit of 0.15 μM was observed for AChE. The electrochemical performance of the biosensor array demonstrated a high selectivity for the simultaneous determination of glutamate and AChE.

Carbon nanotubes (CNTs) are considered hollow carbon structures composed of a rolling graphite sheet, with nanometer scale walls and micrometer scale wall length. Carbon atoms are well arranged in the carbon nanotubes walls via sp2 bonds, which form the stiffest tubes and strongest fibers [20]. According to the layer number of the graphite sheet, the CNTs can be divided into single- walled carbon nanotube (SWCNT) and multi-walled carbon nanotubes (MWCNTs). SWCNT is structured of only a single layer of a seamless molecular cylinder and MWCNT is comprised of more than two layers of graphite sheets [20]. They both possess a unique combination of properties including electrical, magnetic, optical, mechanical, and chemical properties, all of which offer great promise for a wide range of applications. In addition, they provide a large active surface area and an electrocatalytic activity that are advantageous in the development of electrochemical transducers [17,21,22]. Several CNTs and graphene-based NT sensors have been reported [23,24,25] with enhanced catalytic activity, wherein metallic nanoparticles (NPs) are anchored to both graphene and CNT substrate to provide excellent electron mediation for electron transfer between nanoparticles and substrate. Furthermore, NPs are widely used to modify the electrode surface due to their large specific surface areas, excellent conductivities, and catalytic activities. Their unique properties have enabled their use in a multitude of sensors [23,24,26,27]. Several electrochemical transducers incorporating NPs have been reported for the fabrication of NT biosensors [7,28,29].

To enhance the sensitivity, selectivity, and stability of the CV technique for sensing NTs, CNTs have been explored as sensing materials for electrochemical biosensors [17]. CNTs have been demonstrated to promote electron transfer, electrocatalysis and electroanalysis processes because of their considerable mechanical strength, high electrical conductivity, high surface area, good chemical stability, and relative chemical inertness in most electrolyte solutions. A CNT paste electrode modified with sodium dodecyl sulfate (SDS) has been reported for the selective detection of dopamine (DA) in the presence of ascorbic acid (AA) and uric acid (UA) [30]. It was observed that a SDS-modified-CNT paste electrode resulted in higher sensitivity and selectivity towards DA in the presence of AA and UA and exhibited better performance than unmodified electrodes. A linear range of 1 μM–28 μM with a detection limit at 0.33 μM was observed for DA.

Furthermore, alternative electrode fabrication technology involving 3D printing has been used with CNT yarns to develop a new platform for electrochemical sensing of dopamine in the presence of UA and AA [31]. This innovative CNT yarn 3D printed electrode displayed promising electrocatalytic activity for the redox reaction of DA in the presence of AA and UA. The peak potentials were found to be at 50 mV, 305 mV, and 545 mV for AA, DA, and UA, respectively with a DA detection limit of 0.87 ± 0.09 μM. The as-fabricated electrode showed high reproducibility and stability, and most importantly introduced a new versatile platform, compatible with various sensor fabrication techniques including screen-printing. The modification of the electrode surface with nanostructure materials is paramount for the advancement of neurotechnology for the detection of NTs. However, despite the decades of the implementation of CV techniques for the detection of NTs, the technique is limited to in vitro applications and high NT concentrations. In addition, the scan rate is very low (hundreds of millivolts/s) and the temporal resolution must be greatly improved for the detection of NTs *in vivo*.

The detection of NTs *in vivo* can be quite challenging due to the low concentrations and the transient events occurring on the sub-second time scale. Therefore, a faster and more powerful technique is required to detect NT events in real-time. Fast Scan CV (FSCV) is a powerful electrochemical technique which has a fast scan rate of ≥400 V/s, thereby resulting in high temporal resolution. The FSCV technique combines multiple scans taken over time to analyze the changes in neurotransmitter kinetics [32]. The key difference between FSCV and CV is the extremely high scan rate used in FSCV and the millisecond cycle duration (~100 ms). FSCV is suitable for neurotransmitter sensing in vitro and *in vivo* and it is more convenient for *in vivo* applications because of its high temporal resolution when compared to the conventional CV technique. FSCV can be multiplexed and combined with microelectrode arrays to detect various NTs in addition to monitoring the electrophysiology signals in real time, thereby, allowing the study of complex interactions *in vivo* and unraveling the relationship between NTs to local field potentials.

**Figure 3:**
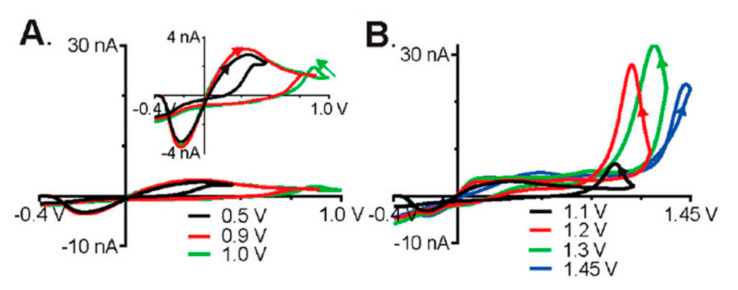
Fast scan cyclic voltammetry (FSCV) of 1 µM histamine at different switching potentials, a holding potential of −0.4 V, and a scan rate of 400 V/s. (**A**) Peak current as the result of electrostatic adhesion of histamine. (**B**) Faradaic peaks upon the reduction of histamine. Reprinted with permission [35].

Probe fouling is a challenge that needs to be overcome in the development of sensing technology. It is the result of reaction byproducts that irreversibly adsorb onto the surface of the probe, thereby negatively impacting the sensitivity of the sensing system. Various approaches have been taken to minimize the biofouling of the electrode surface. Polyhistamine has been shown to foul the surface of microelectrode probes, thereby hindering the detection of histamine [33,34]. Confounding studies on the oxidation potential of histamine have led to in depth analysis on the electrochemical properties of these molecules [35]. FSCV and X-ray photoelectron spectroscopy (XPS) were used to address the conflicting literature proposing an oxidation potential of 1.5 V [33] versus 0.3 V [34] for histamine. Pihel et al. observed that histamine oxidizes at 1.5 V by losing a single proton and single electron and generating a free radical on a nitrogen in the imidizole ring, thereby leading to dimerization and polymerization of histamine. The polyhistamine then electrostatically adheres to the electrode surface, thus fouling the probe. The electropolymerization of the surface proteins was further confirmed with XPS [33]. Nafion coating was explored to minimize the electrode fouling and the results agreed with other works which found a 1.5 V oxidation potential as shown in Figure 3A [35]. Nafion was shown to decrease fouling by reducing histamine adhesion to the surface of the electrode, but as a result, the initial anodic current was reduced. The peak recently seen by Hashemi et al. at 0.3 V was concluded not to be a faradaic peak but change in current caused by surface adhesion of histamine as shown in Figure 3B.

**Figure 4:**
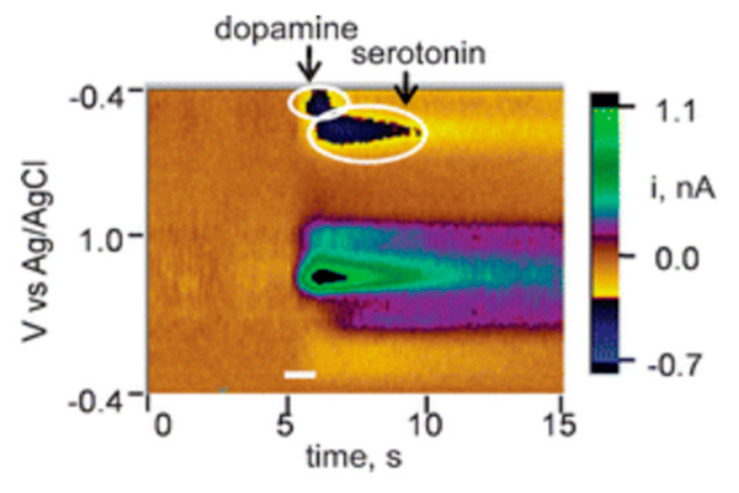
False color plot visualization of the change in concentration of dopamine and serotonin on single carbon nanotube. Reprinted with permission [36].

Serotonin is known also to foul electrode surfaces and as an additional challenge, it has the same oxidation potential as dopamine due to the similarity between the two biomolecules. A single CNT modified carbon fiber electrode was demonstrated *in vivo* to selectively detect serotonin and dopamine in real time as shown in Figure 4 [36]. Simultaneously detection of serotonin and dopamine was achieved by using the reduction potentials. Fouling was addressed by modifying the carbon fiber probe with CNTs, which enhanced the redox peak current generated. The resistance of CNTs to fouling has been observed in other studies, although interestingly the mechanism is not known [37,38].

**Figure 5:**
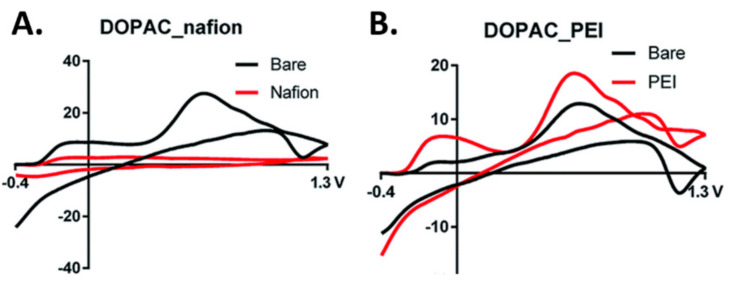
Fast scan cyclic voltammetry scans for 3,4-dihydroxyphenylacetic acid (DOPAC) with nafion (**A**) and polyethyleneimine (PEI) (**B**). Reprinted with permission [39].

3,4-dihydroxyphenylacetic acid (DOPAC), a metabolite of dopamine, has identical oxidation and reduction potentials to dopamine. The structures of DOPAC and dopamine are very similar, except that DOPAC has a carboxyl group located where dopamine has an amine. The presence of this functional group gives DOPAC a negative charge, whereas dopamine has a positive charge at physiological pH. Previously, DOPAC was only measurable using microdialysis and liquid chromatography. However, more recent work has demonstrated that physiologically relevant concentrations of DOPAC can be detected at a polymer modified carbon fiber microelectrode [39]. Nafion coating was used as a polymeric electrode coating to screen out the negative ions and provide a higher affinity for positively charged compounds such as dopamine, whereas polyethyleneimine (PEI) was used to electrostatically attract negatively charged compounds such as DOPAC. Nafion was shown to greatly reduce the signal for DOPAC (Figure 5A) and PEI showed an increase in sensitivity of the signal when compared to the uncoated electrode (Figure 5B). A higher holding potential was used to allow for the preconcentration of DOPAC by varying the holding potentials to include positive, negative, and neutral holding potentials. The optimal holding potential for the detection of DOPAC was determined to be 0 V as opposed to −0.4 V for dopamine with a limit of detection of 52.25 ± 2 nM DOPAC, which was found to be within the physiological range reported by Hashitani et al. [40].

Non-electroactive NTs require the use of enzymes for their detection. One such NT is glutamate. Hunsberger et al. demonstrated *in vivo* glutamate sensing in mice with high spatial resolution [41]. Simultaneous measurements in subregions of the brain were achieved using microelectrode arrays implanted in the dentate gyrus, cornu ammonis 3, and cornu ammonis 1 hippocampal subregions. Glutaraldehyde was used as the crosslinking agent for the immobilization of glutamate oxide onto the microelectrodes [42]. A size exclusion layer of electroplated m-phenylenediamine successfully prevented interference from ascorbic acid and dopamine. High spatial resolution as well as temporal resolution was achieved *in vivo* for glutamate with a linearity of 0 to 20 µM (R^2^ = 0.90) and a limit of detection of 1.5 μM. The *in vivo* detection of glutamate enabled the study of the pharmacological effects on glutamate release and reuptake, as well as the impact on the various regions of the brains. Although the microelectrodes employed in this study achieved high temporal resolution, most electrodes are limited to a temporal resolution of 10 Hz. This can be attributed to an incomplete reverse reaction, causing local concentrations of NT to be converted to the oxidized state.

**Figure 6:**
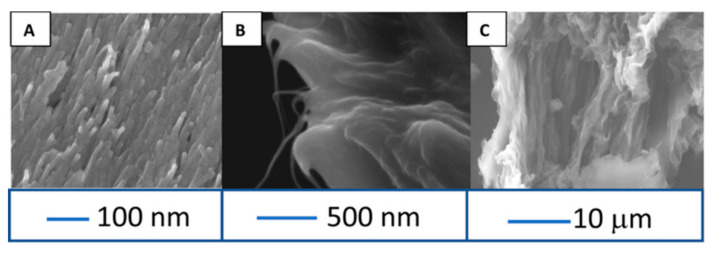
Cross-sectional SEM images of carbon nanotube (CNT) yarn (**A**), PEI-CNT fiber (**B**), and acid spin CNT fiber (**C**). Reprinted with permission [43].

Zestos et al. investigated the physical characteristics and sensing capabilities of fabricated CNT microelectrodes and carbon fiber microelectrodes (CFMEs) and observed a 50× increase in the temporal resolution and 8× increase in the sensitivity of the CNT microelectrodes [43]. These microelectrodes exhibit a high density of carbon edge-planes serving as the catalytic sites for neurotransmitter oxidation. Additionally, the “rough” surface of the electrodes (Figure 6) provided higher catalytic surface area and steric trapping of the NT. The rough surface morphology provided a large surface area for the oxidation of dopamine. The sensing capabilities of a carbon yarn, PEI- CNT fibers, and CFMEs were characterized across a range of scan rates, frequencies, and dopamine concentrations [44,45]. A limit of detection of 24 ± 2 nM, 10 ± 0.8 nM, 3 ± 0.5 μM, and 5 ± 1 μM dopamine were obtained for the unmodified CFME, prefabricated CNT yarn, acid-spun CNT fibers, and PEI-CNT fibers, respectively.

**Figure 7:**
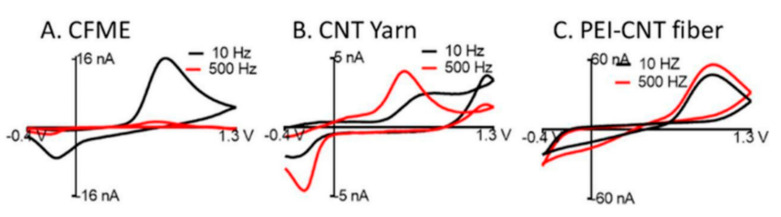
CV of 1 µM dopamine at 2000 V/s for (**A**) carbon fiber microelectrode (CFME) (**B**) CNT yarn, and (**C**) PEI-CNT fiber electrodes, demonstrating a consistent signal even at a high temporal resolution of PEI-CNT fibers. Reprinted with permission [43].

The electrochemical characterization showed that the responses of the fibers were frequency independent up to 500 Hz as shown in Figure 7. Thereby enabling higher temporal resolution as compared to the standard cycle frequencies of CFME (10 Hz) with a scan rate of 400 V/s. The steric trapping of the dopamine quinone on the surface of the fibers was observed to allow for a far more reversible reaction as compared to CFMEs. The high reversibility of the reaction allowed for faster scan rates with higher temporal resolution and potentially may serve as a better electrode platform for studying firing patterns at synaptic junctions *in vivo*.

A persistent limitation of carbon-based microelectrodes for FSCV lies in the fabrication and production of these microelectrodes. The fabrication process requires manual assembly of carbon fiber using the glass pulling technique, which is time consuming, inefficient, and requires a trained technician. This limitation can be improved by using micro-/nanolithography fabrication techniques to produce hundreds of sensors on a single wafer. Recently, a dopamine sensor was developed with over 6000 glassy carbon nanorod probes in a single array [46] using lithography and pyrolysis to enable highly scalable microelectrode fabrication. The fabricated glassy carbon nanorods were similarly as sensitive as a carbon fiber (7 nA/µM vs. 5 nA/µM), with comparable limits of detection (LOD = 60 nM vs. 41 nM), respectively. A linear range of 1–10 µM (R^2^ = 0.9867) dopamine with a sensitivity of 7 ± 3 nA/µM was observed. The fabricated multiprobe array has the potential to provide higher sensitivity, spatial resolution, and ease of fabrication, thereby allowing high throughput detection and monitoring. Though significant advancement has been made to minimize the limitations associated with sensitivity, selectivity, and temporal resolution through the use of FSCV, there still remain an increased demand for new analytical techniques to successfully remove these barriers to NT detection and monitoring to enable the understanding of neurophysiology in clinical research.

### 2.2. Neurosensing via Differential Pulse Voltammetry

Differential pulse voltammetry is a derivative of linear sweep voltammetry or staircase voltammetry, wherein a series of regular voltage pulses are superimposed on the potential linear sweep or stairsteps. This technique is very sensitive and allows for the simultaneous direct analysis of multiple NTs using a single probe with good sensitivity. Wu et al. employed DPV technique for the determination of dopamine (DA) and serotonin (5-HT) using carbon nanotube film-coated glassy carbon electrode (GCE) [47]. It was observed that DA and 5-HT yield two well-defined oxidation peaks at 0.18 V and 0.36 V, respectively. The oxidation peak current increased significantly with the MWCNT coating in contrast to bare GCE. A large excess of interfering analyte, AA resulted in an insignificant influence on the oxidation signals of DA and 5-HT. With a scan rate of 20 mV/s, a linear range of 0.05 µM–5 µM DA and detection limit of 0.011 µM were observed. For 5-HT, the linear range reported was from 0.02 µM to 5 µM with a detection limit of 5 nM. MWCNT-modified electrodes are promising for *in vivo* and in vitro measurements of DA and 5-HT.

**Figure 8:**
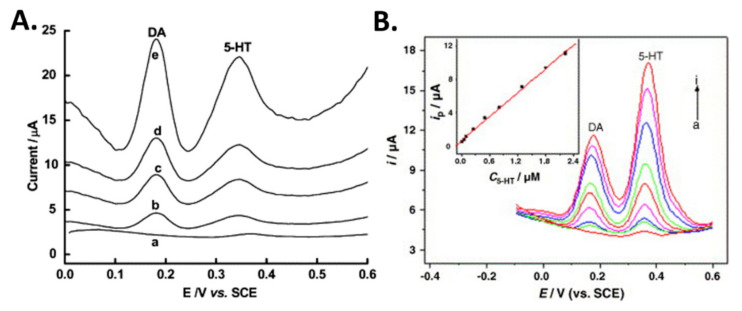
Differential pulse voltammetry (DPV) of dopamine (DA) and serotonin (5-HT) in the human serum conducted using (**A**) MWCNT—DHP film-coated GCE for (a) 0, (b) 0.05, (c) 0.1, (d) 0.2, (e) 0.4 µM DA and 5-HT. (**B**) DPVs of DA + 5-HT at nano-Au/PPyox/GCE with variously spiked concentrations: (a–i) 0, 0.05, 0.1, 0.25, 0.5, 0.8, 1.3, 1.8, 2.2 μM. Reprinted with permission [47,48].

Gold (Au) nanoclusters on insulating overoxidized-polypyrrole (PPyox) GCE (nano- Au/PPyox/GCE) have been shown to improve the detection of dopamine and serotonin [48]. Detection of these analytes in a mixture was possible even at overlapping anodic peaks for 5-HT, DA and AA. The voltammetric peaks were observed at 0.37, 0.20, and 0.01 V by overlapping anodic peaks of 5-HT, DA, and AA (1000-fold), respectively. The linear responses were in the range of 7 nM–2.2 µM with a detection limit of 1.0 nM for 5-HT, and in the range of 0.75 µM–20 µM with a detection limit of 0.15 nM DA. This highlights the electrocatalytic activity of nano-Au/PPyox/GCE towards DA and 5-HT oxidation, wherein an improvement was observed in the overall current response and the oxidation overpotentials were lowered as the result of the synergic effect of the PPyox and Au nanoclusters. A comparative plot of DPVs for the detection of DA and 5-HT in human serum using modified glassy carbon electrodes is shown in Figure 8.

**Figure 9:**
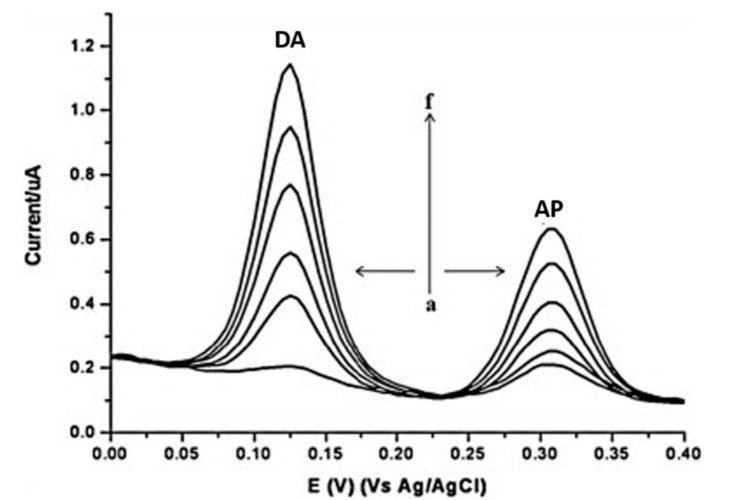
DPV voltammograms of increasing concentrations of DA and AP at a f-MWCNT modified electrode. Reprinted with permission [49].

A lower detection limit for DA was achieved by developing highly sensitive acid functionalized MWCNT modified GCEs for simultaneous electrochemical determination of DA and acetaminophen (AP) in biological fluids [49]. Oxidation peaks at 125 mV and 307 mV were observed for DA and AP, respectively with good stability, reproducibility, repeatability, and high recovery in human serum. The linear dynamic ranges of 3–200 μM DA (R^2^ = 0.992) with a detection limit of 0.8 μM DA and 3– 300 μM AP (R^2^ = 0.989) with a detection limit of 0.6 μM AP were observed. A pre-concentration step was used to allow the accumulation of these species at the electrode surface and subsequently resulted in well separated voltammetric peaks for sensitive and selective determination of DA and AP as shown in Figure 9.

A biosensor array of vertically aligned carbon nanofibers (CNF) [50] was developed using plasma enhanced chemical vapor deposition (PECVD). The CNF electrode was reported to be effective in detection of DA and 5-HT in the presence of AA. The oxidation current was larger for CNF than GCE for individual mixtures of DA/AA and 5-HT/AA. In the ternary mixture of AA, DA, 5-HT, the GCE was unable to distinguish all three species due to overlapping peaks. Thereby, unmodified GCEs are unsuitable for simultaneous detection of NTs. In contrast the CNF electrode can distinguish DA and 5-HT when they co-exist in the same solution. The CNF electrode demonstrated superior sensitivity towards DA, with detection down to 50 nM in comparison to 100 nM for GCE. The CNF electrode shows two linear ranges, from 100 nM–500 nM DA (R^2^ = 0.9996) and from 1 μM–10 μM 5-HT (R^2^ = 0.9603). Both CNF and GCE electrodes detect down to 100 nM for DA. A linear range from 1 μM–10 μM 5-HT (R^2^ = 0.9970) was observed. This suggests CNF electrodes are a great alternative to CNT-modified GCE and graphene-modified GCE.

**Figure 10:**
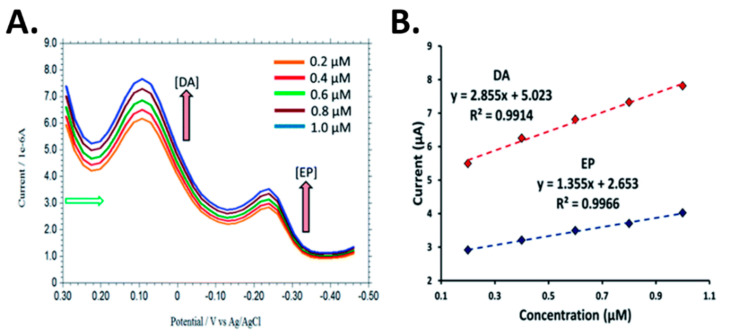
(**A**) DPV voltammograms of increasing concentrations of DA and EP in 0.1 M PBS at AgNPs-PCA-Au electrode. (**B**) Plot of current as a function of concentration of DA and EP. Reprinted with permission [51].

A silver nanoparticles (AgNPs) over penicillamine self-assembled gold electrode [51] was developed for the detection of DA and epinephrine (EP) in the presence of high concentration of AA and uric acid at neutral pH. AgNPs were employed due to their catalytic properties and high electrical conductivity. An enhancement in the electrode sensitivity, selectivity, as well as a wide linear dynamic range with nanomolar detection limit were observed. Chronoamperometry was carried out for the determination of DA and EP in the linear range of 0.1–100.0 mM with detection limits of 0.2 nM DA and 0.5 nM EP. Simultaneous detection of DA and EP was performed using DPV. Figure 10 shows the voltammograms for DA and EP with different concentrations. In the presence of 0.2 mM EP, the peak current of DA was linear from 0.2–1.0 mM.

Graphene is an allotrope of the carbon family with a two-dimensional layer of carbon atoms arranged in a honeycomb-structured network. Graphene has attracted a great deal of attention due to its outstanding and novel properties such as high active surface area, high electrical conductivity, strong mechanical strength, and good chemical stability [52,53,54]. Some graphene-based neurotransmitters have been published [54,55]. Additionally, a microfluidic device has attracted attention in the development of NT biosensing platforms for the purpose of microliter sample detection [55]. A poly(diallyldimethylammonium) chloride-reduced graphene oxide (PDDA-RGO) modified MWCNTs-carbon paste electrode (MWCNT-CPE/PDDA-RGO) was demonstrated to detect dopamine and 5-HT with high sensitivity (0.016 μM and 0.0098 μM), wide linear range (0.05–120.0 μM), good stability (30 day storage), and repeatability for 20 μL sample volume detection [55]. The amalgamation of MWCNT and reduced graphene oxide (RGO) showed promising electrical conductivity, electrochemical stability, and electrochemical performance. The linear ranges obtained were 0.05–120.0 μM DA with limit of detection of 0.016 μM DA (S/N = 3) and 0.05–50.0 μM 5-HT with limit of detection of 0.0098 μM 5-HT (S/N = 3). Good sensitivity and selectivity were observed for dopamine and 5-HT in microliter volume rat’s plasma showing that DPV combined with carbon-based materials has the potential to detect multiple NTs in biological samples at the sample time, although the technique is limited by lower temporal resolution when compared to FSCV. Moreover, concentration changes on the order of minutes can be easily detected using DPV but capturing events that occur on the order of sub-second intervals in real-time requires the use of FSCV.

## 3. *In Vivo* Sensing

Techniques have evolved from developmental to translational research with the establishment of standardized methods for *in vivo* experiments [56,57]. It is important to mention that the suitability of *in vivo* over in vitro NT sensing relies on the specific application. For example, in vitro is suitable for clinical applications, whereas *in vivo* is more applicable for continuous monitoring to detect the state of diseases in a timely manner. However, *in vivo* often give rise to more complex electrodes with lower sensitivity and higher cost. In addition, the techniques with higher sensitivity and reliability are often large, bulky, and require time-consuming manipulations. The NTs released or secreted into the extracellular space by exocytosis can be determine via in vitro and *in vivo* experiments. Carbon fiber microelectrodes and ceramic electrodes are the most commonly used electrodes that can be easily functionalized with enzymes specific for the target NT [57]. For *in vivo* analysis, the electrodes are implanted or semi-implanted in the specific regions of the brain [32]. It has also been reported that ceramic based electrodes functionalized with glutamate oxidase can detect glutamate in *in vivo* rodent and swine models [40,58]. The sub-second temporal resolution of the microelectrodes have allowed monitoring of the release and uptake of glutamate in the hippocampal region of the brain which occurs in milliseconds. The use of validation studies has allowed for in vitro studies to be translated to *in vivo* studies, where NTs have been detected in awake behaving animals including DA, 5-HT, Glu, and AChE [58,59].

**Figure 11:**
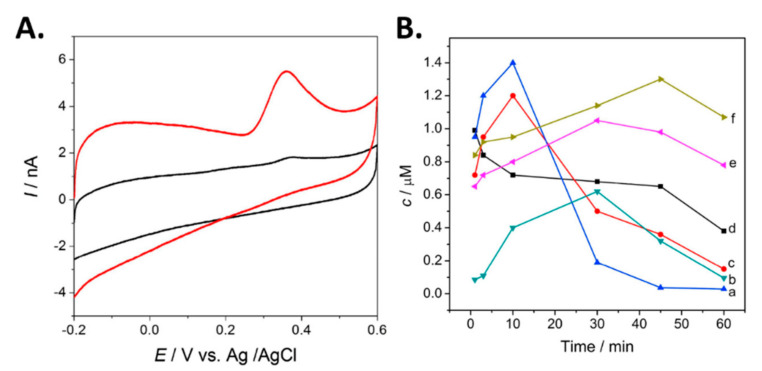
(**A**) CV voltammogram of 5 μM serotonin at the bare microelectrode (black), and the GR- FeTSPc functionalized probe (red). (**B**) Concentration of DA obtained *in vivo* every 10 m with the following experimental conditions: (a) administration group given Uncaria rhyunchophylla, (c) the control administration group given antipsychotics, (d) the negative control group given saline and 5- HT at (b) the negative control group, (e) control administration group, and (f) administration group. Reprinted with permission [60].

DA and 5-HT have been simultaneously detected *in vivo* using DPV [60]. A custom CFME was functionalized with graphene and a metallophthalocyanine to enhance the electrocatalytic capabilities of the electrodes. Figure 11A shows the voltammogram of 5-HT at the fabricated and bare electrodes. An enhanced oxidation peak was observed with the graphene modified CFME. Concentrations of 5-HT and DA were measured simultaneously using DPV at about 10-m intervals one hour after the final dose for the duration of 1 h in mice. Although DPV is a sensitive technique that can simultaneously detect multiple analytes with a single probe, it is limited by its low temporal resolution, as demonstrated by the 10-m intervals shown in Figure 11B, compared to the sub-second intervals of FSCV.

**Figure 12:**
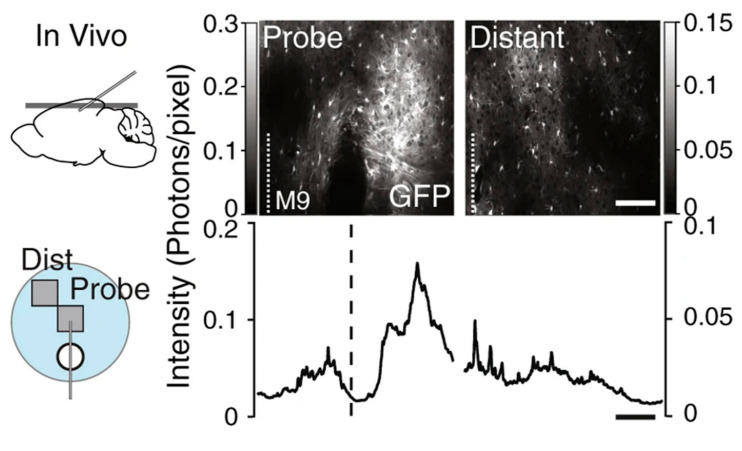
Astrocytes expressing GFP local to probe implantation (probe) and the adjacent area (distant) *in vivo* mice model. An increased density of astrocytes is associated with glial scarring. Reprinted with permission [61].

The current challenges associated with microelectrode probes for the detection of NT include enhancing their longevity, durability, and minimizing tissue damage during implantation. Longitudinal studies have been performed to examine the extent of glial scarring with various probe electrodes [61,62]. The stability and sensitivity of chronically implanted electrodes have been characterized for up to four months [63] and have been utilized in electrochemical characterization *in vivo* using rodent [41], pig [41,64], primate [62], and even human models [65]. Tissue damage during implantation can lead to glial encapsulation of the probe. Parylene [66], silicone [61], and carbon electrodes [63] have been used in chronic implantation, wherein the electrode headstage is fixed to the live animal by performing stereotaxic surgery to target a region in the striatum. When tissue is damaged, glial cells surrounding neurons form a protective barrier, resulting in “glial scarring”. Immunostaining techniques and transgenic reporter animals have been used to visualize the extent of glial scarring, as shown in Figure 12.

Parylene based electrodes of various geometries implanted in rats for over four weeks showed that electrodes less than 12 µm in thickness did not cause glial encapsulation based on qualitative immunostaining [66]. A study using silicone probes investigated the long-term stability of electrophysiology recording and glial scaring in live mice [61]. A consistent reading was obtained for up to 10 weeks post implantation. Transgenic reporter mice are known to express green fluorescent protein (GFP) in astrocytic glial cells (BAC Aldh1l1-GFP+/−) and this was used to visualize the extent of glial scarring. After an initial spike, there was no significant increase in scarring from the second week to the tenth week post implantation. The probe dimensions were 10 mm in length, 100 µm in width, and 50 µm thick. Electrodes with less than 12 µm thickness, it was suggested, can minimize scarring [67]. Regardless of the initial glial scarring observed during the first week (Figure 12), no significant effect on the signal to noise ratio was observed for the duration of the experiment.

Carbon fiber electrodes have been shown to provide stable and consistent readings *in vivo* over a 4-month study on mice [63]. Electrically stimulated and food stimulated DA responses were detected at one, two, and four months after implantation. Immunostaining results showed no loss of signal in either the *in vivo* stimulated DA response, or in the in vitro sensitivity analysis. No gliosis was detectable after a 4-month period, which was attributed to the geometry of the electrode (diameter < 12 µm). However, a higher degree of temporal distortion was observed when compared to acute experiments performed by the same group [63]. Mathematical deconvolution methods have been proposed to address temporal distortion for more precise analysis of long-term studies, such as that developed for primates [62].

**Figure 13:**
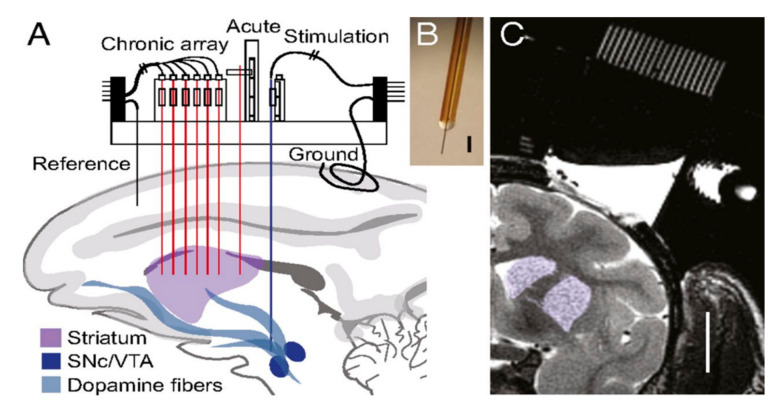
(**A**) A schematic of the implanted electrode. (**B**) Photograph of the implanted working electrode. (**C**) MRI of the striatal target site highlighted in purple. Reprinted with permission [62].

Chronically implanted probes in primates were found to lack structural integrity, where mechanical failure from user handling and sudden primate head movement caused irreparable fractures in the silica insulation of the probe [62]. Three probes failed immediately upon implantation and the rest of the probes failed within 176 days after implantation. However, the behavioral and induced responses to stimulus in various regions of the primate brain for over 100 days were studied [66]. As shown in Figure 13, an array of four working electrodes were implanted in the striatum parallel to acute, reference, and counter electrodes. A stimulation electrode was implanted in the ventral tegmental/substantia nigra to generate electrically stimulated responses. FSCV at a frequency of 10 Hz showed successful neurochemical responses that correlated to behavior and learning.

Deep brain stimulation (DBS) is an established technique used to invoke a dopaminergic response and has been used for the treatment of neurodegenerative diseases, such as Parkinson’s and epilepsy [65]. Although the therapy has been widely successful [67], the underlying mechanisms of DBS are not well understood. Electrophysiology sensing is relatively common during these procedures, where the electrical recordings are collected with the DBS lead continually. Acute neurotransmitter detection in humans has been performed during DBS surgery at the Mayo Clinic by FSCV [65]. FSCV is performed for the duration of the surgical procedure, then the electrode is removed while the DBS replaces the electrophysiology electrode for continuous therapeutic stimulation. FSCV is interpreted using the Wireless Instantaneous Neurochemical Concentration Sensing (WINCS) system. This system generates the waveform, digitizes the electrochemical signal, and transmits the resulting signal to a base station using Bluetooth to enable the investigation of the role of DA in decision making [68]. The next generation of therapeutic electrodes are expected to have wireless electrophysiological and electrochemical sensing capabilities.

## 4. Conclusions

In this review, we summarized the recent development of electrochemical sensors for the detection of NTs. Given the ultralow concentration of NTs (~nM) and the abundance of biomolecules present in the extracellular space, the exact quantification of NTs *in vivo* remains a challenge as NTs are locally generated in a specific region of the brain. With the advancement in nanobiosensing, various carbon, metallic, and polymeric nanocomposite sensing materials have been integrated on microelectrodes for rapid detection of NTs due to their outstanding properties, such as high catalytic activities, electron transfer rates, and high electrical conductivity. These sensing materials have been widely reported to exhibit fast response time, as well as improved sensitivity and selectivity due to their synergistic electrocatalytic activity towards target NTs and the fact that they can be fabricated at lower cost. Polymer materials are widely used for reducing the effects of biofouling and interference from competing and noncompeting analytes and have been shown to be biocompatible. CV, FSCV, and DPV are electrochemical techniques used for the detection of NTs. CV is limited to in vitro applications and required high concentrations of NT and DPV to enable the simultaneous detection of multiple NTs on a single probe, whereas FSCV enables the rapid detection on NTs with high temporal resolution. The temporal resolution on CV is however low compared to FSCV. Therefore, amongst the electrochemical detection techniques discussed, FSCV is the most convenient technique for rapid detection of NT in vitro and *in vivo* as it provides higher temporal and spatial resolution. Non-electroactive NTs are detected with a biorecognition element such as an immobilized enzyme on the electrode surface. However, the complex immobilization procedure, enzyme denaturation overtime, and the lack of simultaneous multianalyte detection pose significant challenges for enzyme-based biosensors.

Future advancements are focused on the development of high throughput miniaturized probe arrays using micro-/nanofabrication techniques and the integration of nanostructured sensing materials to improve the long-term stability, sensitivity, selectivity, and reproducibility for rapid diagnostic and point-of-care detection of NTs in real-time. Paired with electrophysiology and DBS, modern techniques can be developed to provide new modalities for neuroscience, medical treatment, and translational research. Extensive validation studies have culminated in providing meaningful insights from *in vivo* studies that show inconsequential effects of tissue damage and allow for the development of ground-truth based signal sorting algorithms. Wireless communication of electrochemical signals generated from FSCV allows a high degree of freedom, which is important for removing restrictions on head movement as an extraneous variable for studying psychological effects and will lead to the reduction physical damage to the electrode or subjects during *in vivo* studies.
